# Tunable High-Pressure Field Operating on a Cationic Biphenyl Derivative Intercalated in Clay Minerals

**DOI:** 10.1038/s41598-017-08064-0

**Published:** 2017-08-09

**Authors:** Makoto Tominaga, Yukihiro Nishioka, Seiji Tani, Yasutaka Suzuki, Jun Kawamata

**Affiliations:** 1 Graduate School of Medicine, Yamaguchi University, 1677-1 Yoshida, Yamaguchi-shi Yamaguchi, 753-8512 Japan; 2 Research Fellow of Japan Society for the Promotion of Science (JSPS), Kojimachi, Chiyoda-ku Tokyo, 102-0083 Japan; 3 Graduate School of Sciences and Technology for Innovation, Yamaguchi University, 1677-1 Yoshida, Yamaguchi-shi, Yamaguchi, 753-8512 Japan

## Abstract

We propose a methodology for applying a pseudo uniaxial pressure to an organic molecule under ordinary temperature and pressure, namely by intercalation into smectites. The pseudo pressure on a biphenyl derivative (BP) was estimated from the averaged dihedral angle around the central bond of BP. In a high hydrostatic pressure field, biphenyl takes a planar conformation. In the interlayer space of synthetic saponite (SSA), the averaged dihedral angle of BP at a loading level of 27% versus the cation exchange capacity was ~26.3°, which indicates that the pseudo pressure applied to BP in the SSA interlayer space corresponds to 0.99 GPa. The high pseudo-pressure field in the interlayer space of SSA was also confirmed by absorption measurements. The dihedral angle around the central bond of the biphenyl moiety decreased to enhance the planarity of the molecule, mainly in response to the electrostatic force that operates between the negatively charged SSA layer and the interlayer cation. The pseudo pressure operating on BP in the smectite interlayer space could be controlled by varying the smectite layer charge density and/or the BP loading level. By using this methodology, controllable pseudo high-pressure properties of organic molecules can be obtained at ordinary temperatures and pressures.

## Introduction

A high-pressure field alters the stereostructure of molecules in solution. For example, the dihedral angle around the central bond that connects the two phenyl rings in biphenyl decreases from 45° to 20° when the hydrostatic pressure increases from ordinary pressure to 1.2 GPa^1^. Such a stereochemical change produces various interesting features, including enhanced reactivity because of a shortening of the bond length^2–4^, expansion of the π-electron system because of an improvement in the molecular planarity^1, 5^, and generation of radical species through bond cleavage^6, 7^. Similar stereostructural changes occur when a molecule is encapsulated in a microscopic cavity in an inorganic host^8, 9^. For example, the Raman spectrum of oxygen molecules that are adsorbed in a metal–organic framework corresponds to that observed at 2 GPa^9^. In addition, tetrakis(1-methylpyridinium-4-yl)porphyrin is flattened in the smectite interlayer space, whereas the pyridinium ring is twisted with respect to the plane of the porphyrin ring in solution because of steric hindrance^10, 11^. The interlayer space thus causes molecules to adopt a similar flattened conformation to that observed in a high-pressure field.

Smectites are a group of layered inorganic clay minerals^12^. Each layer is negatively charged because of isomorphous substitution by lower-valence cations. The negative charges are compensated by exchangeable inorganic cations, which are commonly hydrated in the interlayer spaces. These cations are readily exchanged by other inorganic or organic cations^9^. Consequently, the exchanged cations are consequently confined in the smectite interlayer space.

The principle force that flattens a cationic organic molecule in the interlayer space of a smectite is the electrostatic force between the exchangeable inorganic cations and/or intercalated organic cations and the anionic sites of the smectite layer^12^. Because the electrostatic force applies to the vertical direction of a smectite layer plane, the force is uniaxial, and the uniaxial force per unit area becomes stronger as the charge density of the smectite increases. When a molecule with a thickness larger than the gallery height of the smectite is intercalated under specific conditions (which depend on the type of intercalated species, humidity and other factors), the uniaxial force applies to the molecule. We regard this uniaxial force as a pseudo uniaxial pressure. It should be noted that the uniaxial force is not applied to molecules with thicknesses smaller than the gallery height of the smectite.

As described above, the uniaxial force per unit area is determined by the type of smectite. The uniaxial force applied to intercalated molecules is shared among all molecules existing in the unit area. Therefore, the uniaxial force, that is, the pseudo uniaxial pressure applied to one molecule, decreases as the number of molecules per unit area increases. Consequently, it is possible for intercalated organic molecules to experience a tunable pseudo-high-pressure field by varying the charge density or loading level.

**Figure 1:**
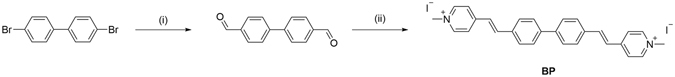
Synthesis and chemical structure of BP. (i) *n*-BuLi, *N,N′*-dimethylformamide, THF, −78 °C, (ii) 1,4-dimethylpyridinium iodide, piperidine, chloroform/methanol (1:3), 60 °C.

We investigated the pseudo pressure as applied to a biphenyl derivative (BP) confined in the smectite interlayer space. The pressure was calculated with a reported equation^1^ that relates the biphenyl dihedral angle to the pressure. The average dihedral angle about the central bond of a biphenyl moiety in a BP that is confined in the smectite interlayer space was estimated from the molecular thickness that corresponds to the gallery height estimated by X-ray diffraction (XRD). We designed a cationic biphenyl derivative, 4,4′-[(1,1′-Biphenyl)-4,4′-diyldi-(1*E*)-2,1-ethenediyl]bis[1-methylpyridinium] diiodide (BP), with a strong electronic absorption at a visible wavelength (Fig. 1) as the guest organic molecule, and monitored the spectral shift of the absorption band that was induced by changes in the stereostructure. For comparison, the absorption spectrum of BP in methanol at an isotropic hydrostatic pressure was measured with a diamond anvil cell.

## Results

### Gallery heights of hybrid films that consist of BP and smectites

**Figure 2:**
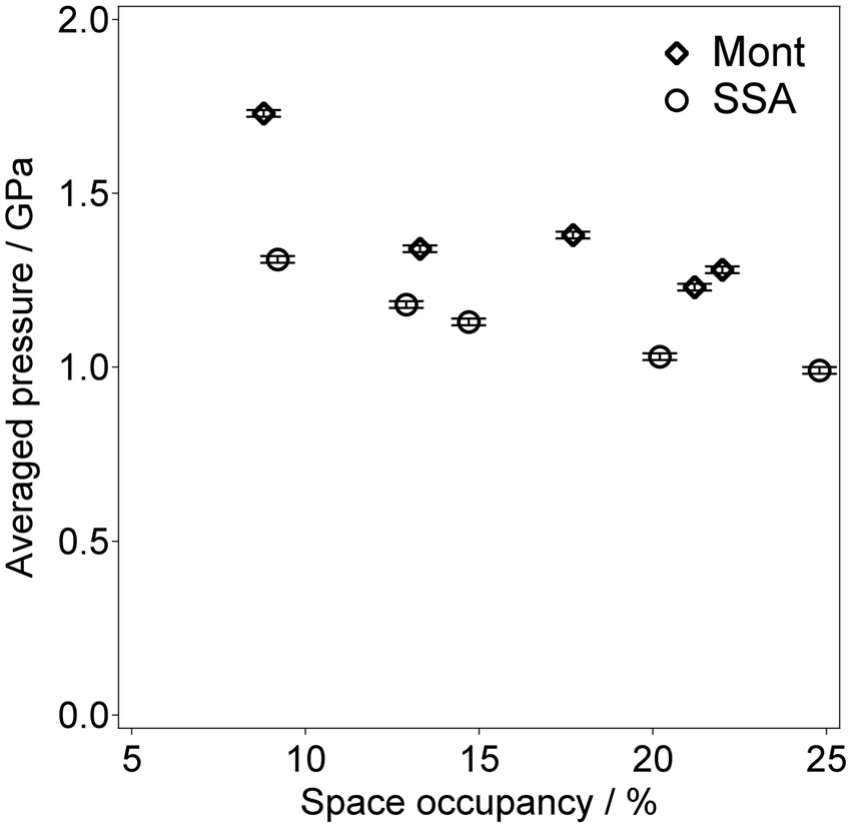
Dependence of averaged pressure on space occupancy of BP in SSA or Mont interlayer space. Diamonds and circles indicate results for hybrid films with Mont and SSA, respectively.

Gallery heights of hybrid films fabricated at various loading levels of BP versus the cation exchange capacity (CEC), which indicates the amount of exchangeable cations per unit weight, with synthetic saponite (SSA) or montmorillonite (Mont) as host smectites were estimated by XRD. Table 1 lists the %CEC, the BP space occupancy, the gallery height as estimated by XRD, the average BP dihedral angle as calculated from the molecular thickness that corresponds to the gallery height, and the corresponding pressure as determined using Eq. (1)^1^, which relates the biphenyl dihedral angle to the pressure:

1 $$P=0.053({\theta }_{0}-\theta ){\rm{G}}{\rm{P}}{\rm{a}}$$

where *P* is the pressure, *θ*_0_ is the dihedral angle at ordinary pressure (45°) and *θ* is the given dihedral angle. BP was assumed to be incorporated in the smectite with its π-plane almost parallel to the clay-layer surface. Space occupancy is defined as the percentage of the interlayer space that is occupied homogeneously by BP molecules^10^. Because the partial exchange of cations in smectites always occurs in a segregated manner and produces random interstratification^13^, a large variation in the space occupancy occurs at each interlayer space in the hybrid films. Table 1 lists average values for various BP states in a hybrid film. Figure 2 shows the averaged pseudo pressure as a function of space occupancy. Mont-BP hybrid films tended to show a higher averaged pseudo pressure than those of the SSA-BP hybrid films. For both the Mont-BP and SSA-BP hybrid films, the averaged pseudo pressure decreased with increasing space occupancy.

**Table 1:** Gallery heights, averaged BP dihedral angles and corresponding pressures applied to BP in hybrid films fabricated at various space occupancies.

	%CEC	Space occupancy (%)	Gallery height (nm)	Averaged dihedral angle (degree)	Corresponding pressure (GPa)
Mont-BP	8.3	8.8	0.453 (1)	12.4 (1)	1.73 (1)
	12.5	13.3	0.505 (1)	19.8 (1)	1.34 (1)
	16.7	17.7	0.499 (1)	18.9 (1)	1.38 (1)
	20.0	21.2	0.520 (1)	21.8 (1)	1.23 (1)
	20.8	22.0	0.513 (1)	20.9 (1)	1.28 (1)
SSA-BP	10.0	9.2	0.509 (1)	20.3 (1)	1.31 (1)
	14.0	12.9	0.526 (1)	22.7 (1)	1.18 (1)
	16.0	14.7	0.533 (1)	23.7 (1)	1.13 (1)
	22.0	20.2	0.546 (1)	25.6 (1)	1.03 (1)
	27.0	24.8	0.550 (1)	26.3 (1)	0.99 (1)

### BP in solution at ordinary and high pressure, and in the smectite interlayer space

**Figure 3:**
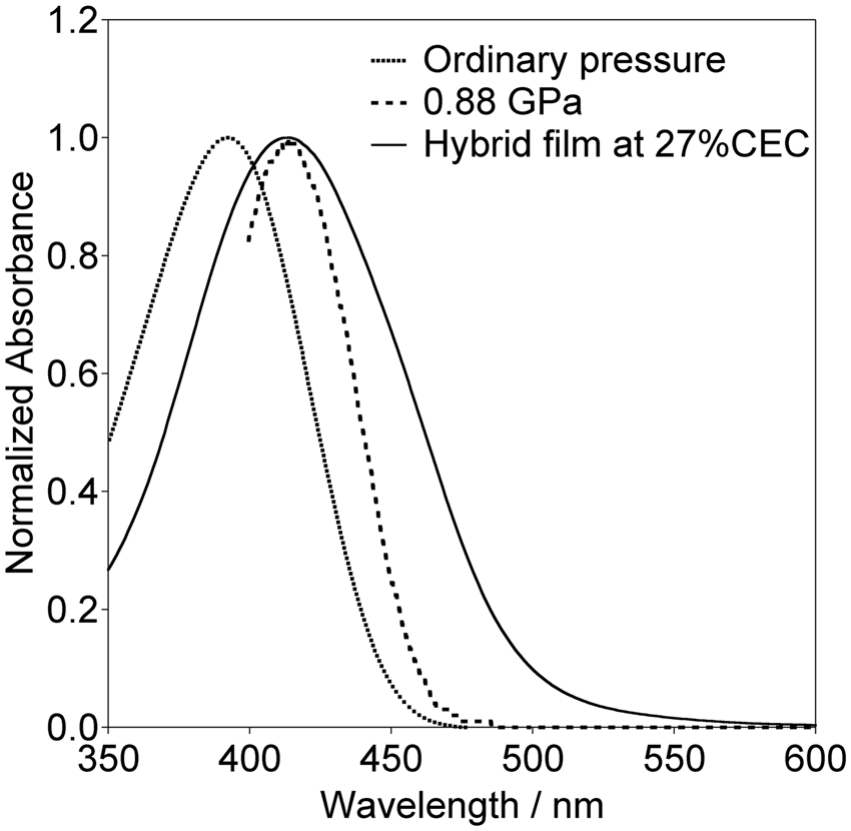
Absorption spectra of BP in a hybrid film fabricated at 27% CEC, and in solution at ordinary pressure and at 0.88 GPa. The BP spectrum at 0.88 GPa could not be measured below 400 nm because of absorption by the window of the diamond anvil cell.

Figure 3 shows the electronic absorption spectra of the SSA-BP hybrid films and those in solution at ordinary and high pressure. The hybrid film was fabricated at 27% CEC and the solution was a methanol solution that contained 1.0 × 10^−4^ mol dm^−3^ BP at ordinary pressure and a hydrostatic pressure of 0.88 GPa. The absorption spectrum of the SSA-BP hybrid film that was fabricated at 27% CEC was red-shifted relative to that for BP in solution at ordinary pressure. The wavelength of the BP absorption peaks in the hybrid film was almost the same as that in solution at 0.88 GPa.

### Pseudo uniaxial pressure field predicted by the Coulomb model

There is an electrostatic force between the interlayer cations and the anionic layer sites. We estimated the pressure applied to BP from the Coulombic force between an interlayer cation and an anionic site of a smectite layer, and assumed that BP was homogeneously adsorbed on the clay layer. The Coulombic force between a cation and an anionic site was estimated. For an SSA-BP hybrid film fabricated at 27% CEC, the interlayer distance, which is the sum of the gallery height and the SSA layer thickness, was 1.51 nm. The distance between the interlayer cation and the anionic site was 1.51/2 = 0.76 nm. Thus, the attractive Coulombic force was calculated to be 4.0 × 10^−10^ N (2 × *q*_1_ *q*_2_/4π*ε*_*o*_ *r* ^2^ = 2 × 0.5 × (1.60 × 10^−19^) × (1.60 × 10^−19^)/4 × π × 8.85 × 10^−12^ × (0.76 × 10^−9^)^2^). A charge of 0.5*e* was used for the charge of an anionic site because the anionic sites in the SSA layer interact with cations on both sides of the layer. The attractive force was doubled because the interlayer cations interact with anions in the upper and lower layers in the hybrid film. The repulsive Coulombic force that operates between anionic sites in neighbouring layers was calculated to be 2.5 × 10^−11^ N (*q*_1_ *q*_2_/4π*ε*_*o*_ *r* ^2^ = 0.5 × (1.60 × 10^−19^) × 0.5 × (1.60 × 10^−19^)/4 × π × 8.85 × 10^−12^ × (1.51 × 10^−9^)^2^). Consequently, a total Coulombic force of 3.8 × 10^−10^ N per anionic site is considered to be applied to an interlayer species in the interlayer space. The pressure applied to BP was estimated by dividing the Coulombic force by the area of BP. The surface area of the SSA per anionic site is estimated to be 1.25 nm^2^, and 27% CEC corresponds to a 24.8% space occupancy in an SSA-BP hybrid. Therefore, it was estimated that a pseudo uniaxial pressure of 1.2 GPa ( = 3.8 × 10^−10^/(1.25 × 10^−18^ × 0.248)) was applied to BP molecules in the SSA interlayer space. The calculated value is of the same order of magnitude as the observed 0.88 GPa. Thus, the magnitude of the pseudo pressure as estimated by this Coulomb model is consistent with the experimental results.

## Discussion

**Figure 4:**
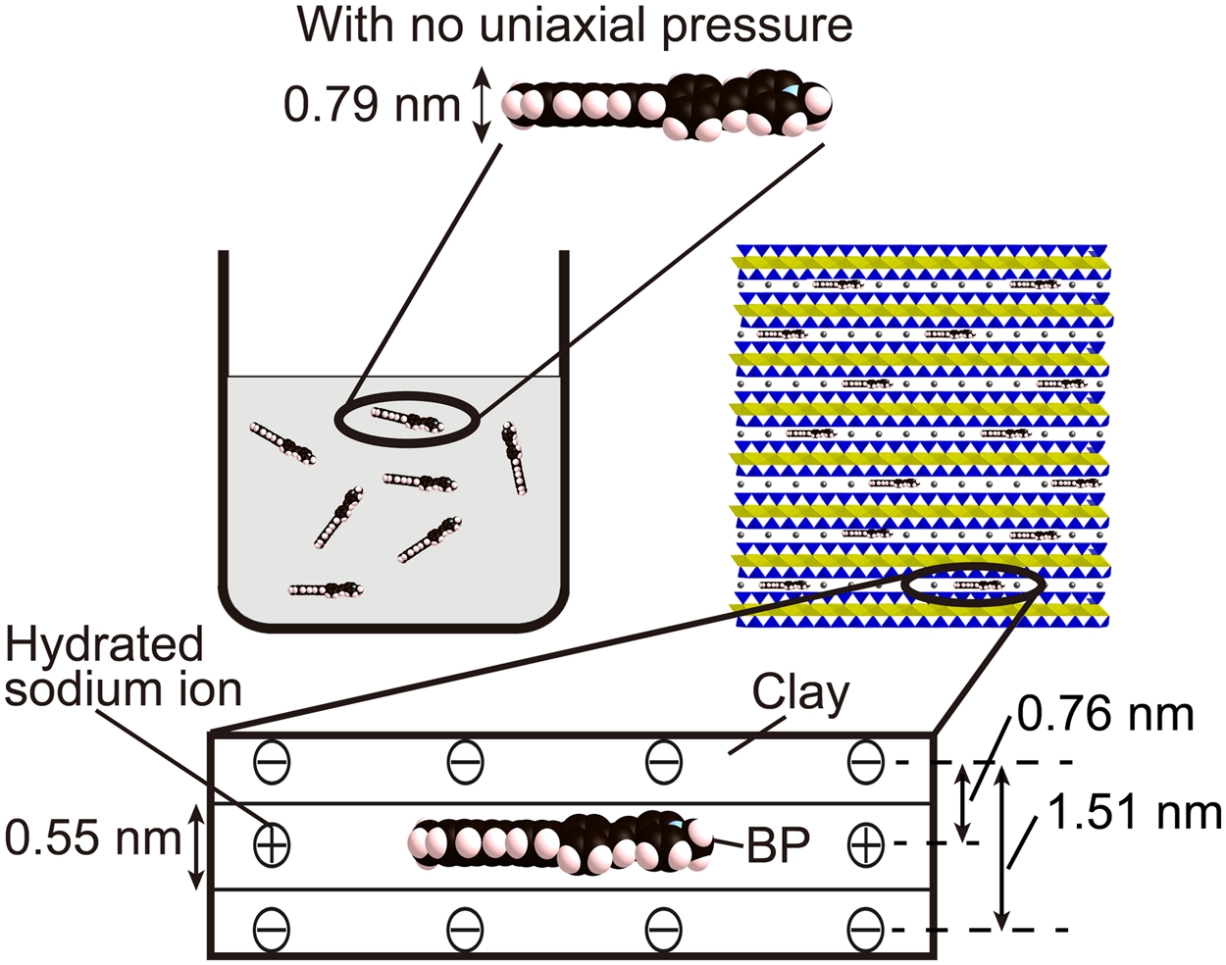
Schematic representation of molecular confinement of BP in solution and in a hybrid film fabricated at 27% CEC. Sodium ions and anion sites in the smectite are indicated by circles that contain plus and minus signs, respectively.

The gallery height of an SSA-BP hybrid film fabricated at 27% CEC was estimated to be 0.55 nm by XRD, as shown in Table 1. This value is smaller than the molecular thickness (0.79 nm) of BP with a dihedral angle of 45°, that is, its form in solution at ordinary pressure (see Fig. 4). The obtained gallery height corresponds to a dihedral angle of 26°, which means that the planarity of BP is improved in the SSA interlayer space. Biphenyl takes a planar conformation at high pressure^1^. Thus, the molecular conformation of BP in the SSA interlayer space is similar to its conformation in a high-pressure field.

Figure 2 indicates that the averaged pseudo pressures of the Mont-BP hybrid films tended to be higher than those of the SSA-BP hybrid films. The charge density of Mont is higher than that of SSA, and thus, we can consider that the principle force applied in the interlayer space is electrostatic. The van der Waals force between BP and a clay surface may also be one possibility of origin of uniaxial force operating on BP. We estimated the van der Waals force operating between BP and a clay surface based on values reported for that between an organic molecule and a silicate surface^14^. As a result, the contribution was comparable only when BP takes a completely flat conformation and all of the carbon atoms in BP have contacted with clay surface. However, the results of XRD measurement indicated that the BP molecules treated in this study is still distorted even at the lowest %CEC. This means some carbon atoms in BP could not contact with a clay surface. For example, when the %CEC was 27, the average distance between the center of atoms in BP and a clay surface is ca. 1.5 times larger compared with the case that all of the atoms in BP are in contact with a clay surface. Considering the fact that the Coulomb force is proportional to the square of the distance between atoms whereas the van der Waals force is proportional to the sixth power of the distance, the assumption that the principle force applied to BP in the interlayer space is the Coulomb force seems to be reasonable. The averaged pseudo pressure decreases with increasing space occupancy for SSA and Mont hybrids. This trend is explained by considering the increased number of BP molecules in the interlayer space. As the space occupancy is increased, the electrostatic force per unit area is shared by a larger number of BP molecules, which lowers the averaged pseudo pressure.

As shown in Fig. 3, the wavelength of the BP absorption peak observed for a hybrid film fabricated at 27% CEC and that for a methanol solution of the BP at 0.88 GPa were identical (414 nm). However, a wider absorption band was observed for the hybrid film than for BP in methanol at 0.88 GPa. There is a large variation in space occupancy at each interlayer space because the partial exchange of cations in smectites occurs in a segregated manner^13^. The wider absorption band may be attributed to this inhomogeneous confinement, and thus indicates the different microenvironments of the BP in the intercalation state. The high mobility of the intercalated BP compared with that at a high hydrostatic pressure may be other possible reason.

The wavelengths of the absorption peak for SSA-BP hybrid films that were fabricated at 10% and 1% CEC were 422 nm and 429 nm, respectively. The wavelengths were red-shifted compared with that of the 27% CEC SSA-BP as the BP loading level was decreased. The most probable reason for the red shift is a decrease in the dihedral angle around the central bond of the biphenyl moiety, namely, an enhancement of its planarity; this is supported by DFT and TD-DFT calculations, as shown in the Supplementary Information.

In conclusion, this study demonstrates that molecules that are intercalated in the smectite interlayer space undergo a conformational change when the thickness of the intercalated molecule is larger than the gallery height of the smectite. This conformational change depends on the loading level and/or layer charge of the smectite, and can be attributed to a pseudo-uniaxial pressure field that is caused by an electrostatic force that operates between the smectite layer and the interlayer cations. The magnitude of the pseudo pressure field is of the order of a few GPa and can be controlled by varying the layer charge density and/or the space occupancy. This methodology provides a means to tune the stereochemical properties of an intercalated molecule, and induces similar changes to those observed in a high-pressure field. The intercalated molecules experience a pseudo high-pressure field even under ordinary pressure conditions. Thus, the intercalation of organic molecules into the smectite interlayer space is a novel and simple strategy for obtaining the high-pressure properties of organic molecules.

## Methods

### Synthesis of BP

We designed a BP bearing a stilbazolium moiety as a cationic guest molecule with strong electronic absorption at a visible wavelength. 4,4′-[(1,1′-Biphenyl)-4,4′-diyldi-(1*E*)-2,1-ethenediyl]bis[1-methylpyridinium] diiodide (BP) was synthesized as shown in Fig. 1.

### Synthesis of 4,4′-diformylbiphenyl

To a solution of 4,4′-dibromobiphenyl (1.56 g, 5 mmol) in THF (40 ml) was added a 2.6M solution of *n*-butyllithium in hexane (20 ml) dropwise under argon at −78 °C. The reaction mixture was stirred at this temperature for 20 min, and then *N,N′*-dimethylformamide (5 ml) was added dropwise to the reaction mixture. The mixture was allowed to warm gradually to room temperature and was stirred for 3 h. After quenching the reaction with 4 N hydrochloric acid (45 ml), the organic layer was extracted with toluene (3 × 15 ml) and dried over MgSO_4_. The solvent was evaporated, and the residue was recrystallized from hexane to give 4,4′-diformylbipheny in 40% yield. ^1^H NMR (400 MHz, CDCl_3_, ppm), δ = 10.12 (s, 2 H, -C*H*O), 8.03 (d, J* = *8.4 Hz, 4 H, biphenyl), 7.83 (d, J* = *8.0 Hz, 4 H, biphenyl).

### Synthesis of BP

To a solution of a 4,4′-diformylbiphenyl (0.21 g, 1 mmol) in a mixed solvent (50 ml) of chloroform/methanol (1:3) was added six drops of piperidine. The reaction mixture was stirred for 6 h at 60 °C. The precipitate was filtered and then recrystallized from methanol to give BP in 50% yield. ^1^H NMR (400 MHz, DMSO-*d*_6_, ppm), δ = 8.88 (d, J* = *6.4 Hz, 4 H, pyridyl), 8.24 (d, J* = *6.8 Hz, 4 H, pyridyl), 8.07 (d, J* = *16.0 Hz, 2 H, ethenyl), 7.94 (d, J* = *8.4, 4 H, biphenyl), 7.88 (d, J* = *8.4, 4 H, biphenyl), 7.60 (d, J* = *16.4 Hz, 2 H, ethenyl), 4.27 (s, 6 H, -C*H*_3_). Anal. Calcd for C_28_H_26_N_2_I_2_: C, 52.19; H, 4.07; N, 4.35. Found: C, 51.97; H, 4.14; N, 3.47.

### Clay minerals

Synthetic saponite (SSA, Smecton SA, Kunimine Industries, Japan) and sodium montmorillonite (Mont, Kunipia F, Kunimine Industries, Japan) were obtained from the Clay Science Society of Japan. The CEC values of SSA and Mont were 0.997 and 1.15 meq g^−1^, respectively^15, 16^. They were used as received without any purification or pretreatment.

### Fabrication of hybrid films

Hybrid films that consisted of clay minerals and BP were fabricated according to the method in our previous paper^17, 18^. Clay-BP hybrids were prepared by an ion-exchange reaction by mixing aqueous dispersions of clay minerals with a dimethylsulfoxide solution of BP. Hybrid films were prepared by filtering the dispersions of Clay-BP hybrids under suction through a mixed cellulose ester membrane filter (Advantec, A010A025A; pore size: 100 nm; diameter: 25 mm). The films on the membrane filter were transferred to a glass substrate. The loading level, described as the %CEC of organic molecules, in a clay-BP hybrid film was defined as the ratio of BP to clay mineral CEC. The %CEC of the clay-BP hybrid films was controlled by varying the BP concentration.

### Measurements of ultraviolet–visible spectra

The absorption spectrum of a methanol solution of BP was measured under an ordinary pressure of 1.0 × 10^−4^ mol dm^−3^ with an ultraviolet–visible spectrometer (JASCO, U-670) using a 10-mm cuvette. The same spectrometer equipped with an attachment for films (JASCO, VTA-752) was used to obtain the film absorption spectra. The absorption spectrum of the methanol solution of BP was measured under a high pressure of 1.0 × 10^−4^ mol dm^−3^ in a diamond anvil cell with type-IIa diamonds (Syntek Co., Ltd., Japan). BP methanol solution (1.0 × 10^−4^ mol dm^−3^) was introduced with graphite tips into a circular hole of an aluminium gasket with a 400-μm hole diameter. A tungsten halogen lamp (Ocean Optics, LS-1-LL) was used as the light source, and the transmitted light was detected with a USB spectrometer (Ocean Optics, USB4000). The internal pressure in the diamond anvil cell was determined by the previously reported method^19^.

### X-ray diffraction

The interlayer spacing of each hybrid film was determined by XRD. XRD patterns were obtained using a Rigaku Ultima-IV diffractometer with monochromatized Cu Kα radiation (λ = 0.154 nm). XRD measurements of the hybrid films were conducted at 50% relative humidity.

## Electronic supplementary material

Tunable High-Pressure Field Operating on a Cationic Biphenyl Derivative Intercalated in Clay Minerals
