# Supplementary material for: Tunable High-Pressure Field Operating on a Cationic Biphenyl Derivative Intercalated in Clay Minerals
Source: Sci Rep. 2017 Aug 9;7:7651. doi: 10.1038/s41598-017-08064-0 (PMC5550449; doi:10.1038/s41598-017-08064-0)
Supplement: Supplementary file 1 — Tunable High-Pressure Field Operating on a Cationic Biphenyl Derivative Intercalated in Clay Minerals [file 41598_2017_8064_MOESM1_ESM.pdf]

Tunable High-Pressure Field Operating on a Cationic Biphenyl Derivative Intercalated in Clay Minerals

Makoto Tominaga[1,2], Yukihiro Nishioka[1], Seiji Tani[3], Yasutaka Suzuki[1,3], Jun Kawamata[1,3,*]

[1]Graduate School of Medicine, Yamaguchi University, Yoshida, Yamaguchi 753-8512, Japan

[2]Research Fellow of Japan Society for the Promotion of Science (JSPS), Kojimachi, Chiyoda-ku, Tokyo 102-0083, Japan

[3]Graduate School of Sciences and Technology for Innovation, Yamaguchi University, Yoshida, Yamaguchi 753-8512, Japan

Correspondence and requests for materials should be addressed to J.K. (email: j_kawa@yamaguchi-u.ac.jp)

Density functional theory calculations. All density functional theory[1] (DFT) calculations were performed with the Gaussian 09 program package[2]. The geometry of BP in the ground state (S_0) was fully optimized in vacuo using the hybrid functional B3LYP[3,4] in conjunction with the 6-31G(d,p) basis set[5-8]. The absorption properties of BP at the optimized geometry in vacuo were obtained using the time-dependent density functional theory[9-15] (TD-DFT) method at the B3LYP/6-31+G(d,p) level of theory. The five lowest-energy singlet excited states were calculated. From the harmonic vibrational analysis of the optimized BP in S_0 it was confirmed that the optimized geometry is a stationary point that corresponds to the energy minimum on the potential energy surface of S_0. Absorption wavelengths were calculated while changing the dihedral angle from 0° to 60° around the central bond of the biphenyl moiety in BP. Surfaces of molecular orbitals associated with the electronic transitions were generated and visualized with GaussView 5.0.9 (Gaussian, Inc.) The surfaces of the highest occupied molecular orbital (HOMO) and lowest unoccupied molecular orbital (LUMO) of BP with the optimized geometry (twisted conformation) and a planar conformation are shown in Figs. S1 and S2, respectively. Absorption ($S_0 \rightarrow S_1$) wavelengths for all dihedral angles around the central bond of the biphenyl moiety in BP are shown in Fig. S3. The absorption wavelength is red-shifted as the dihedral angle of BP decreases. This tendency is similar to that observed experimentally.

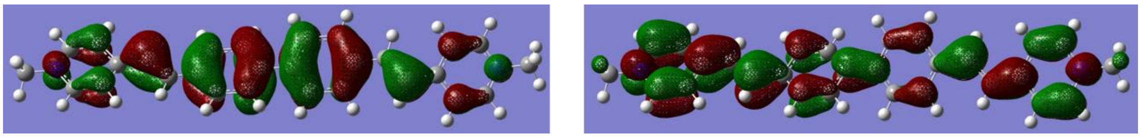

Figure S1. Surfaces of the highest occupied molecular orbital (HOMO, left) and lowest unoccupied molecular orbital (LUMO, right) of BP with the optimized geometry calculated in vacuo at the B3LYP/6-31+G(d,p) level.

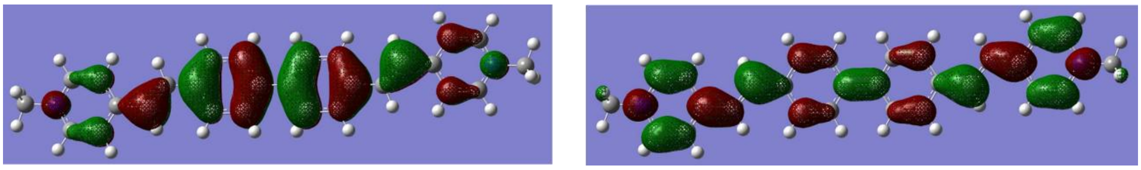

Figure S2. Surfaces of the highest occupied molecular orbital (HOMO, left) and lowest unoccupied molecular orbital (LUMO, right) of BP with a planar conformation calculated in vacuo at the B3LYP/6-31+G(d,p) level.

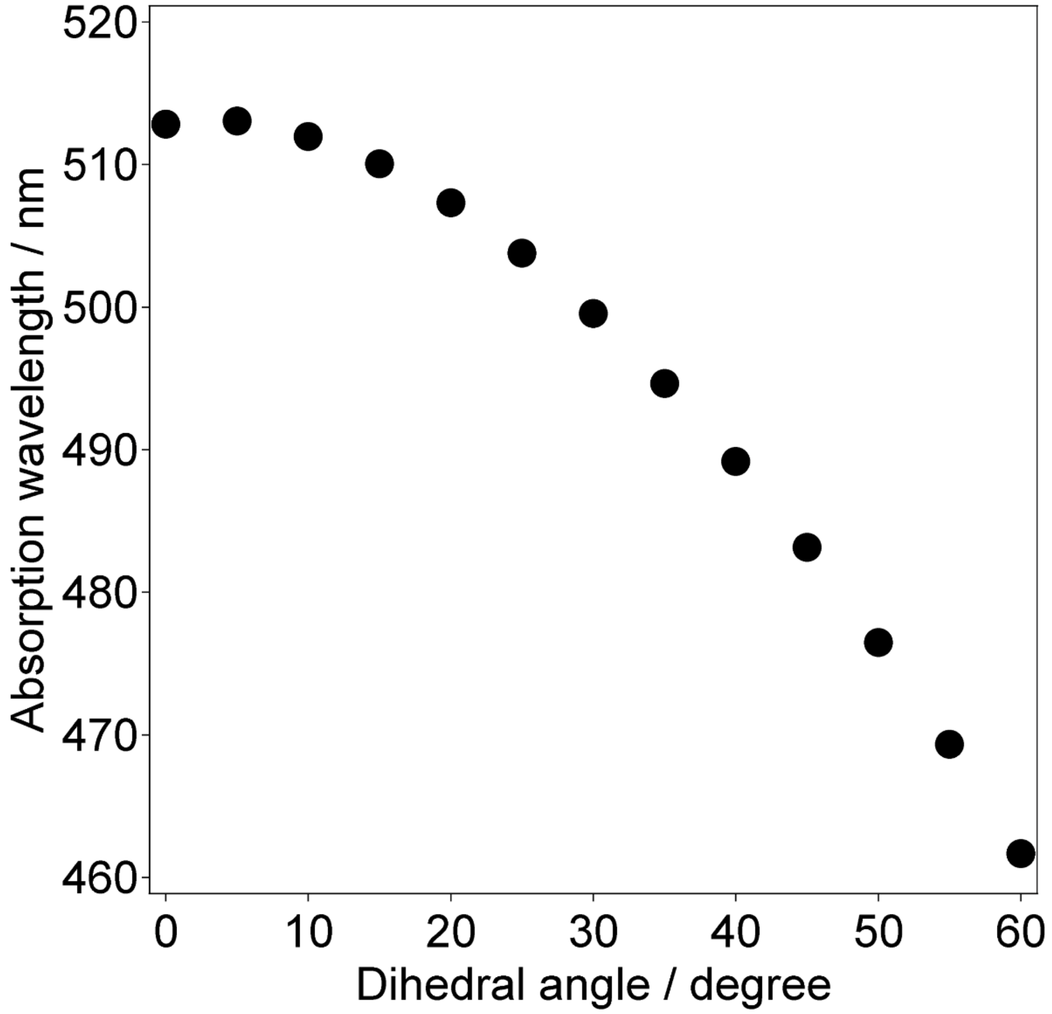

Figure S3. Absorption wavelengths of BP for all dihedral angles around the central bond of the biphenyl moiety in BP.

Fluorescent properties of SSA-BP hybrid films. The fluorescence wavelengths of the SSA-BP hybrid films were measured with an absolute photoluminescence quantum yield measurement system (Hamamatsu Photonics, C9920-02G). The absorption and fluorescence wavelengths, wavenumbers and Stokes shifts are shown in Table S1. The Stokes shift increased as the loading level increased. This indicates that the molecular planarity decreased as the loading level increased.

Table S1. Absorption and fluorescence wavelengths, wavenumbers and Stokes shifts for SSA-BP hybrid films

%CEC	Absorption		Fluorescence		Stokes shift(cm^{-1})
	Wavelength (nm)	Wavenumber (cm^{-1})	Wavelength (nm)	Wavenumber (cm^{-1})	
10	422	23697	551	18149	5548
14	420	23810	550	18182	5628
16	420	23810	553	18083	5727
22	417	23981	566	17668	6313
27	414	24155	578	17301	6854

References

1. Kohn, W. & Sham, L. J. Self-Consistent Equations Including Exchange and Correlation Effects. *Phys. Rev.* **140**, 1133-1138 (1965).

2. Frisch, M. J. *et al*. Gaussian 09, rev. D.01; Gaussian, Inc.: Wallingford, CT (2013).

3. Becke, A. D. Density-Functional Thermochemistry. III. The Role of Exact Exchange. *J. Chem. Phys.*

98, 5648-5652 (1993).

4. Lee, C., Yang, W. & Parr, R. G. Development of the Colle−Salvetti Correlation-Energy Formula into a Functional of the Electron Density. *Phys. Rev. B: Condens. Matter Mater. Phys.* **37**, 785-789 (1988).

5. Ditchfield, R., Hehre, W. J. & Pople, J. A. Self-Consistent Molecular-Orbital Methods. IX. An Extended Gaussian-Type Basis for Molecular-Orbital Studies of Organic Molecules. *J. Chem. Phys.* **54**, 724-728 (1971).

6. Hehre, W. J., Ditchfield, R. & Pople, J. A. Self-Consistent Molecular Orbital Methods. XII. Further Extensions of Gaussian-Type Basis Sets for Use in Molecular Orbital Studies of Organic Molecules. *J. Chem. Phys.* **56**, 2257-2261 (1972).

7. Hariharan, P. C. & Pople, J. A. The Influence of Polarization Functions on Molecular Orbital Hydrogenation Energies. *Theor. Chem. Acc.* **28**, 213-222 (1973).

8. Clark, T., Chandrasekhar, J., Spitznagel, G. W. & Schleyer, P. V. R. Efficient Diffuse Function-Augmented Basis Sets for Anion Calculations. III. The 3-21+G Basis Set for First-Row Elements, Li−F. *J. Comput. Chem.* **4**, 294-301 (1983).

9. Bauernschmitt, R. & Ahlrichs, R. Treatment of Electronic Excitations Within the Adiabatic Approximation of Time Dependent Density Functional Theory. *Chem. Phys. Lett.* **256**, 454-464 (1996).

10. Casida, M. E., Jamorski, C., Casida, K. C. & Salahub, D. R. Molecular Excitation Energies to High-Lying Bound States from Time-Dependent Density-Functional Response Theory: Characterization

and Correction of the Time-Dependent Local Density Approximation Ionization Threshold. *J. Chem. Phys.* **108**, 4439−4449 (1998).

11. Stratmann, R. E., Scuseria, G. E. & Frisch, M. J. An Efficient Implementation of Time-Dependent Density-Functional Theory for the Calculation of Excitation Energies of Large Molecules. *J. Chem. Phys.* **109**, 8218−8224 (1998).

12. Van Caillie, C. & Amos, R. D. Geometric Derivatives of Excitation Energies using SCF and DFT. *Chem. Phys. Lett.* **308**, 249−255 (1999).

13. Van Caillie, C. & Amos, R. D. Geometric Derivatives of Density Functional Theory Excitation Energies using Gradient-Corrected Functionals. *Chem. Phys. Lett.* **317**, 159−164 (2000).

14. Furche, F. & Ahlrichs, R. Adiabatic Time-Dependent Density Functional Methods for Excited State Properties. *J. Chem. Phys.* **117**, 7433−7447 (2002).

15. Scalmani, G. *et al*. Geometries and Properties of Excited States in the Gas Phase and in Solution: Theory and Application of a Time-Dependent Density Functional Theory Polarizable Continuum Model. *J. Chem. Phys.* **124**, 094107−094121 (2006).
